# Supplementary material for: RNA Binding Protein PTBP1 Promotes the Metastasis of Gastric Cancer by Stabilizing PGK1 mRNA
Source: Cells. 2024 Jan 12;13(2):140. doi: 10.3390/cells13020140 (PMC10814388; doi:10.3390/cells13020140)

File S1

Uncropped blots for Figure 1C

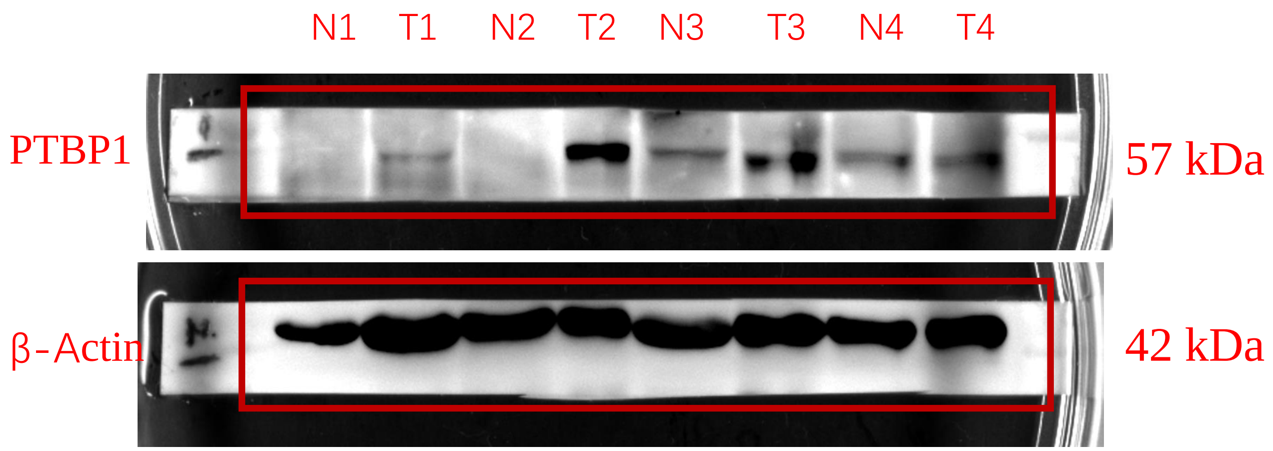

Uncropped blots for Figure 1D

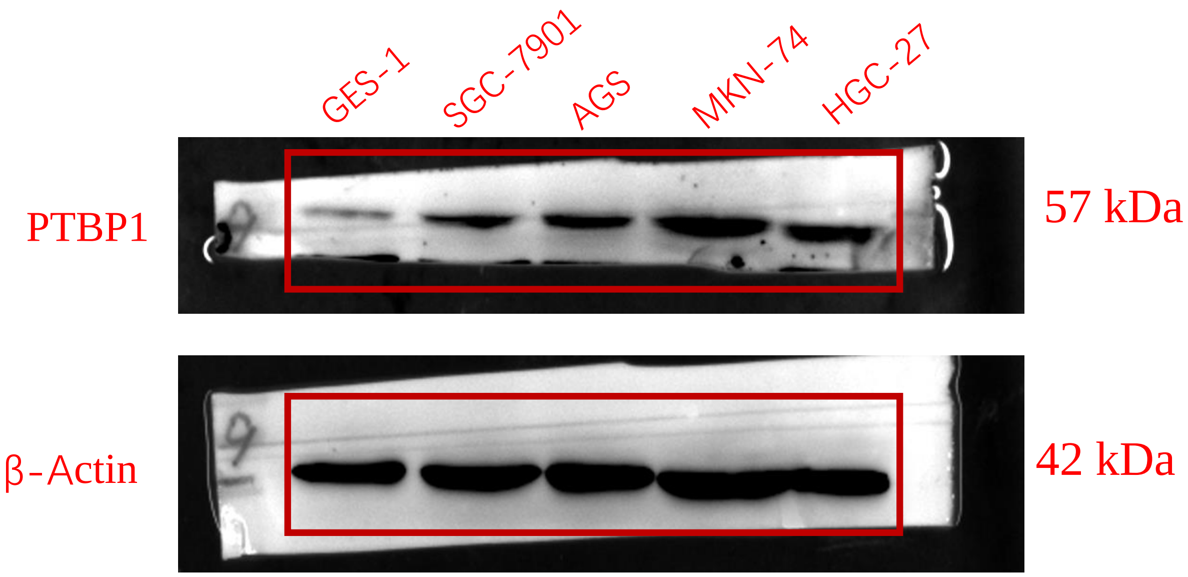

File S1

Uncropped blots for Figure 1J (AGS)

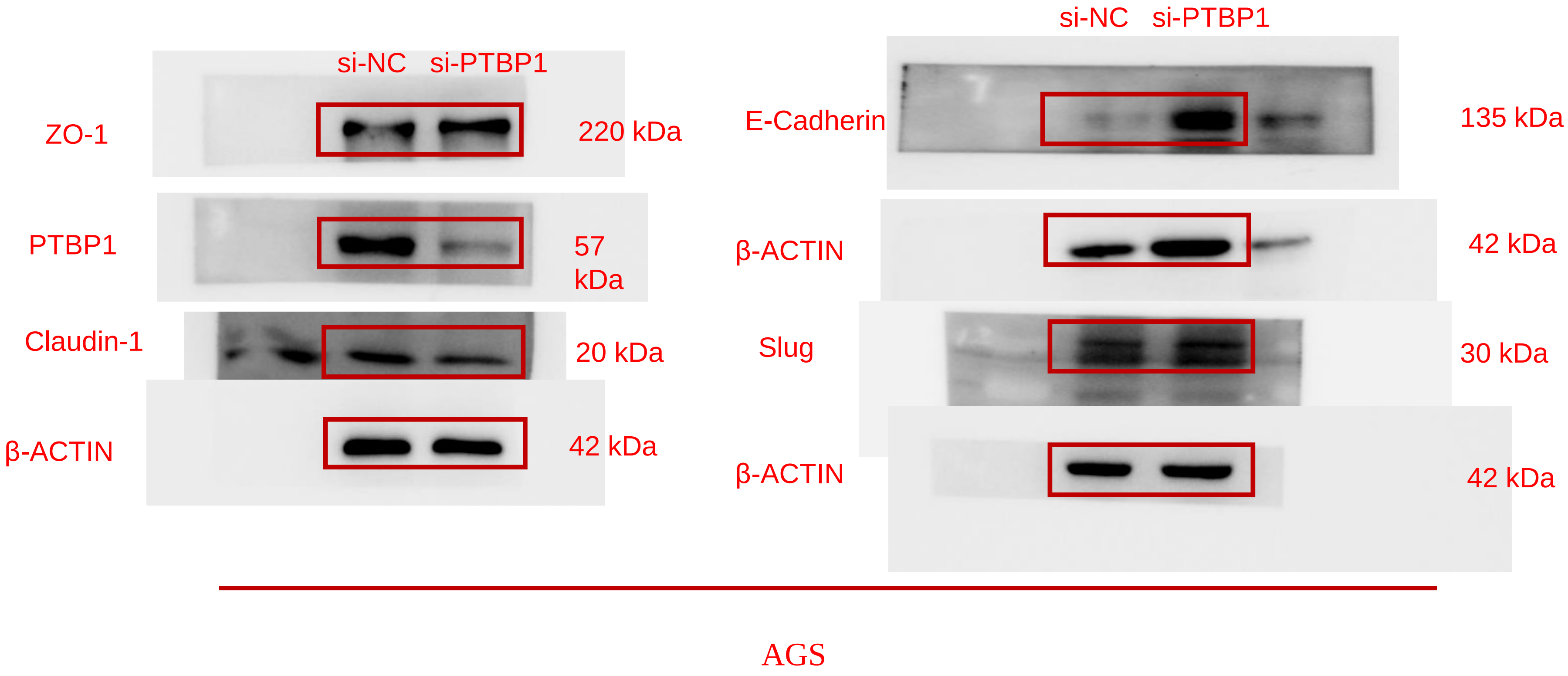

File S1

Uncropped blots for Figure 1J (HGC-27)

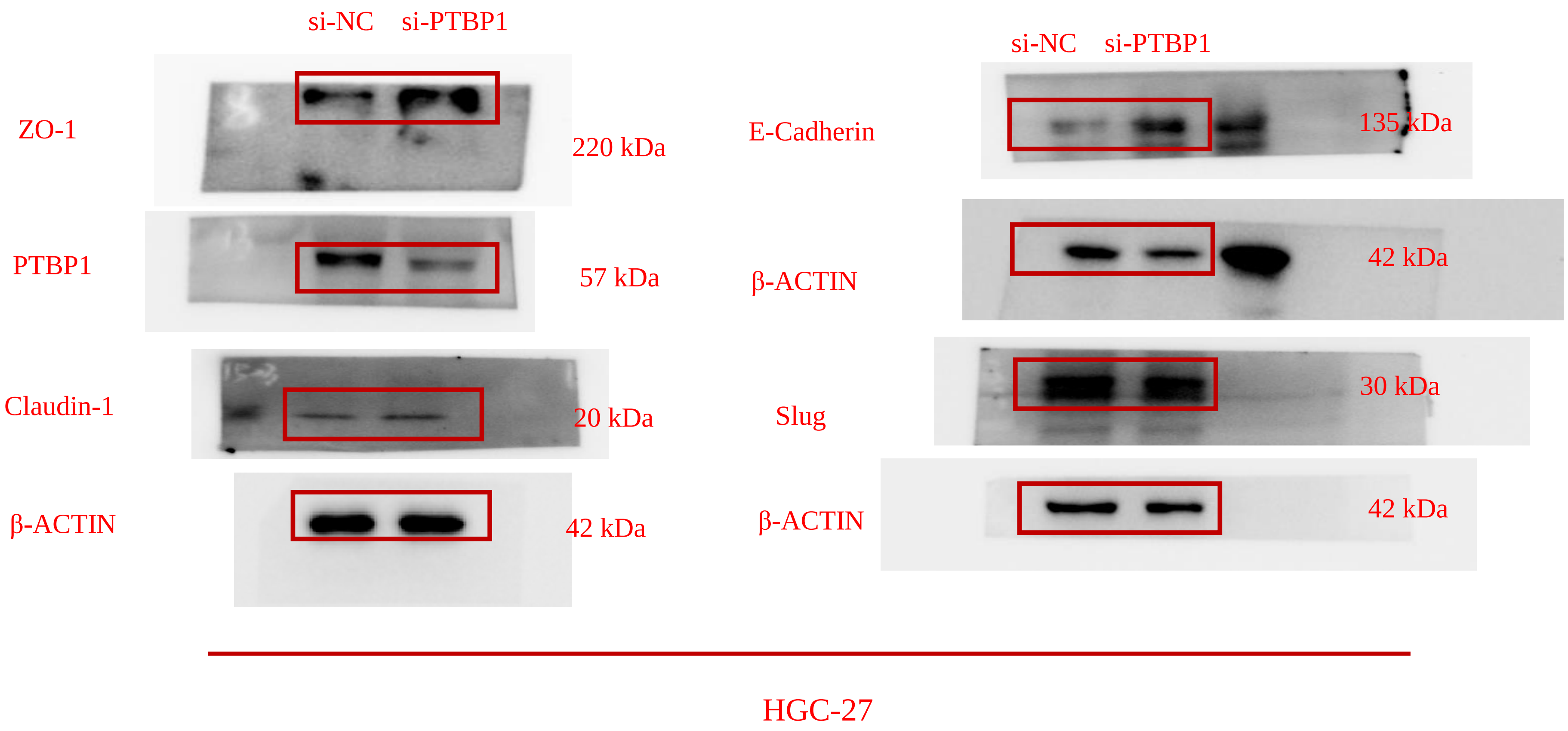

File S1

Uncropped blots for Figure 2B

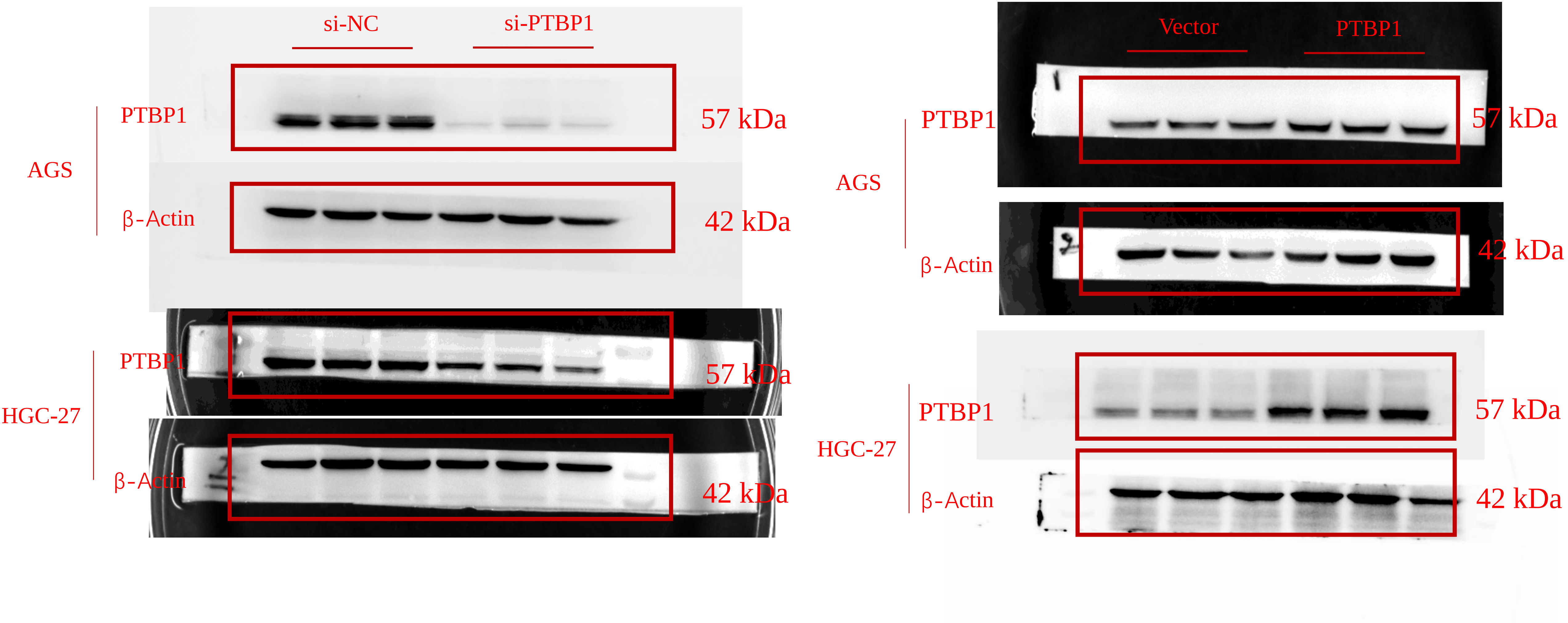

File S1

Uncropped blots for Figure 2E

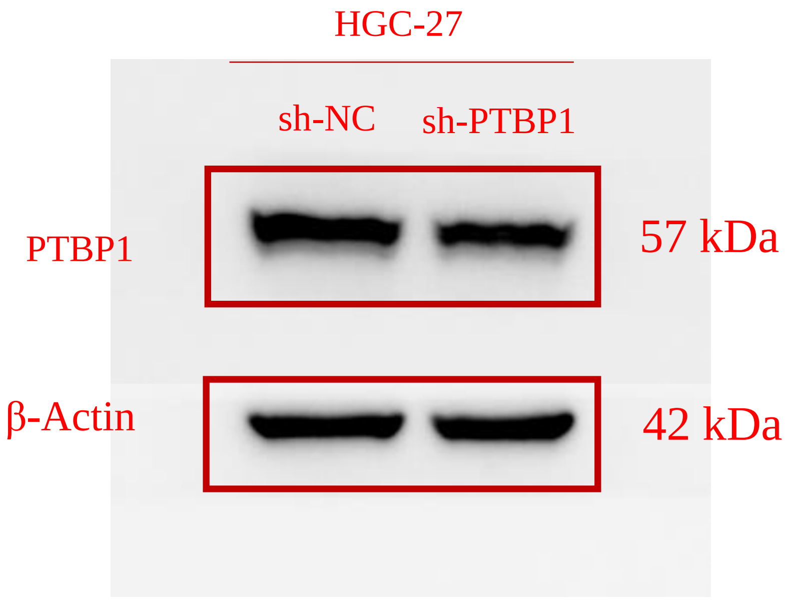

File S1

Uncropped blots for Figure 3G

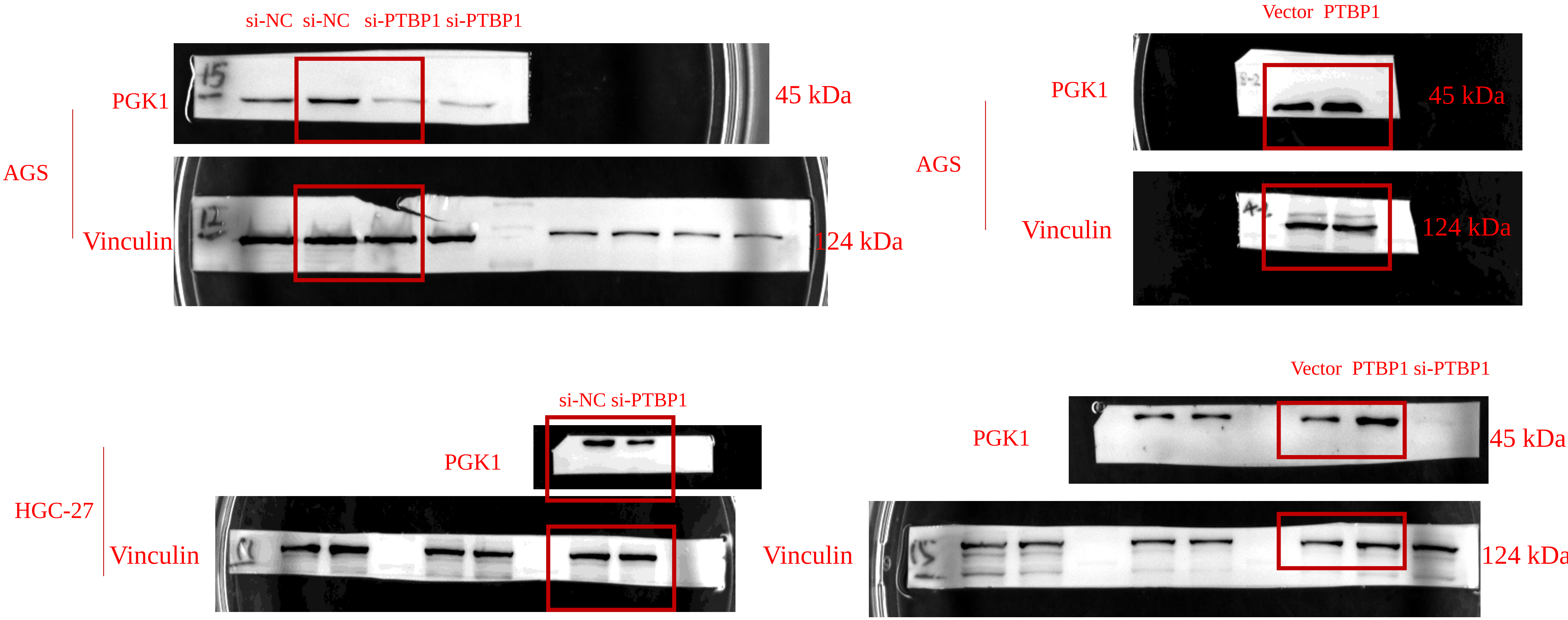

File S1

Uncropped blots for Figure 4A

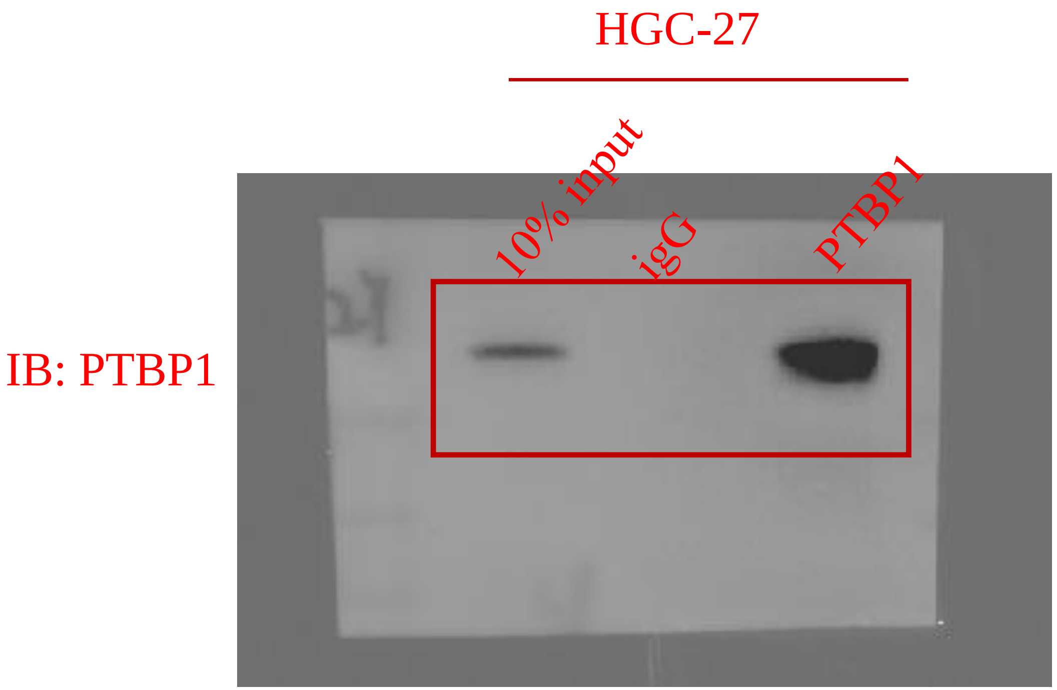

Uncropped blots for Figure 4F

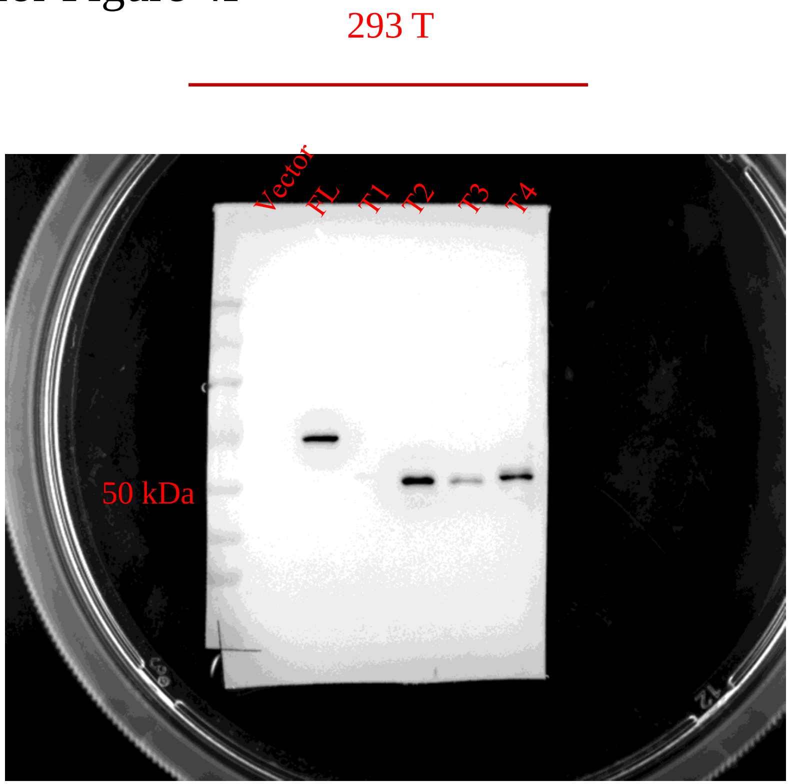

File S1

Uncropped blots for Figure 4G

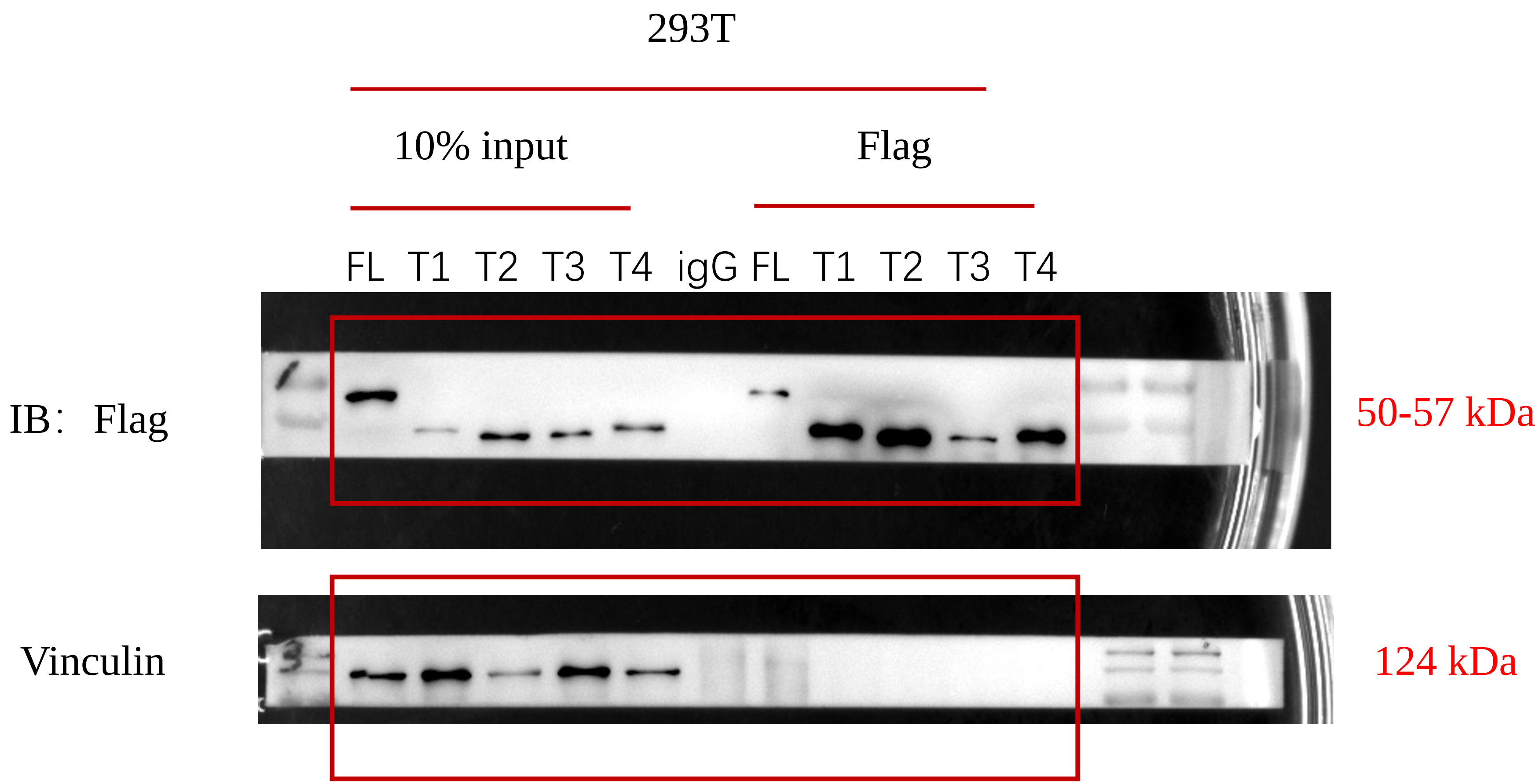

File S1

Uncropped blots for Figure 5C

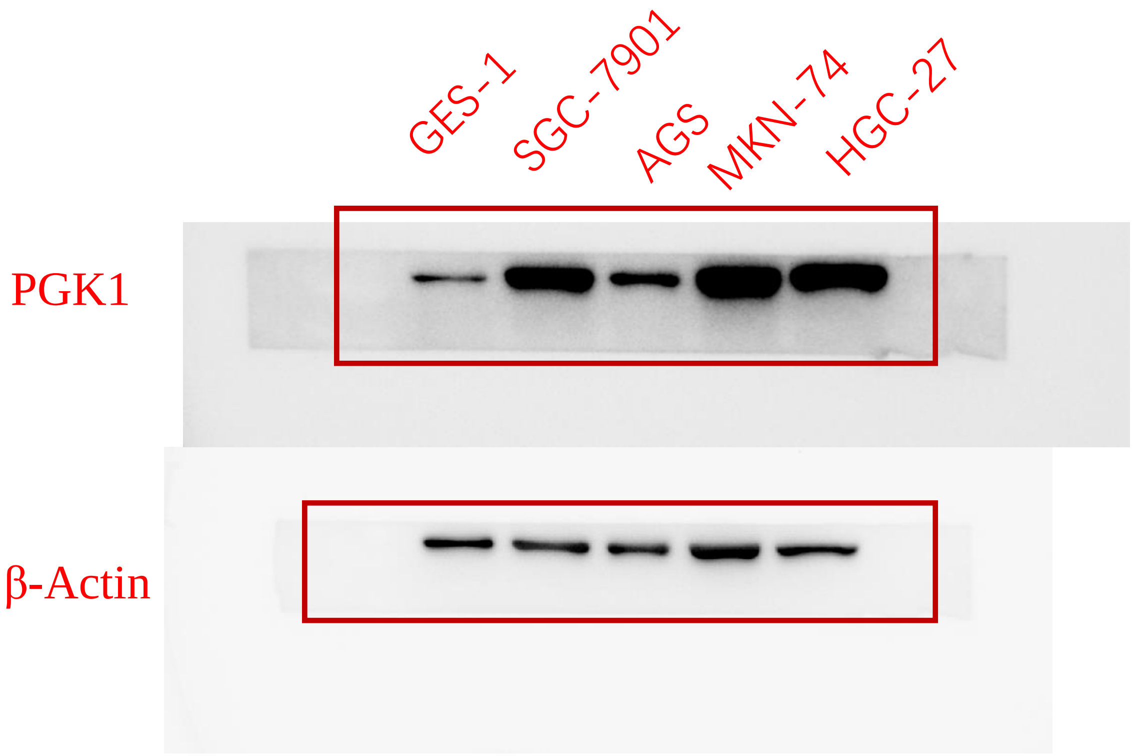

Uncropped blots for Figure 5G

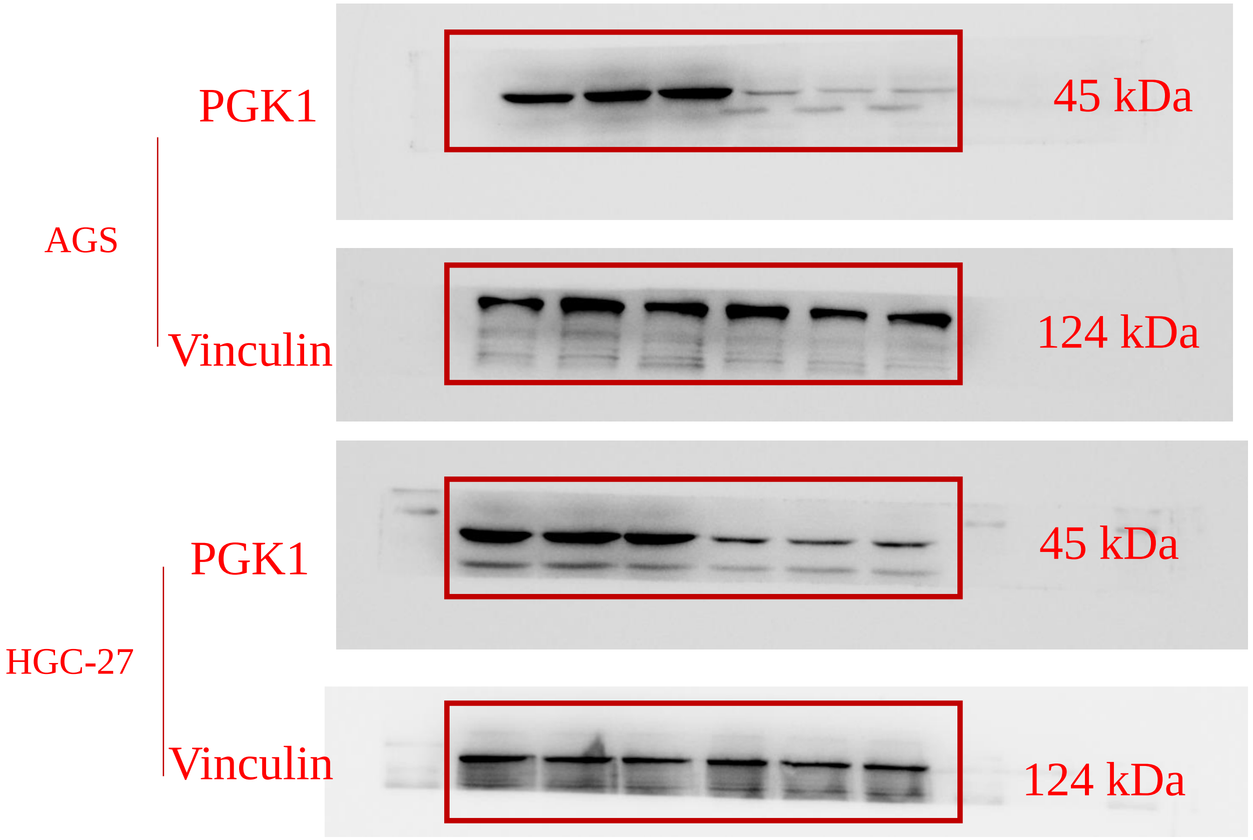

File S1

Uncropped blots for Figure 6A (AGS)

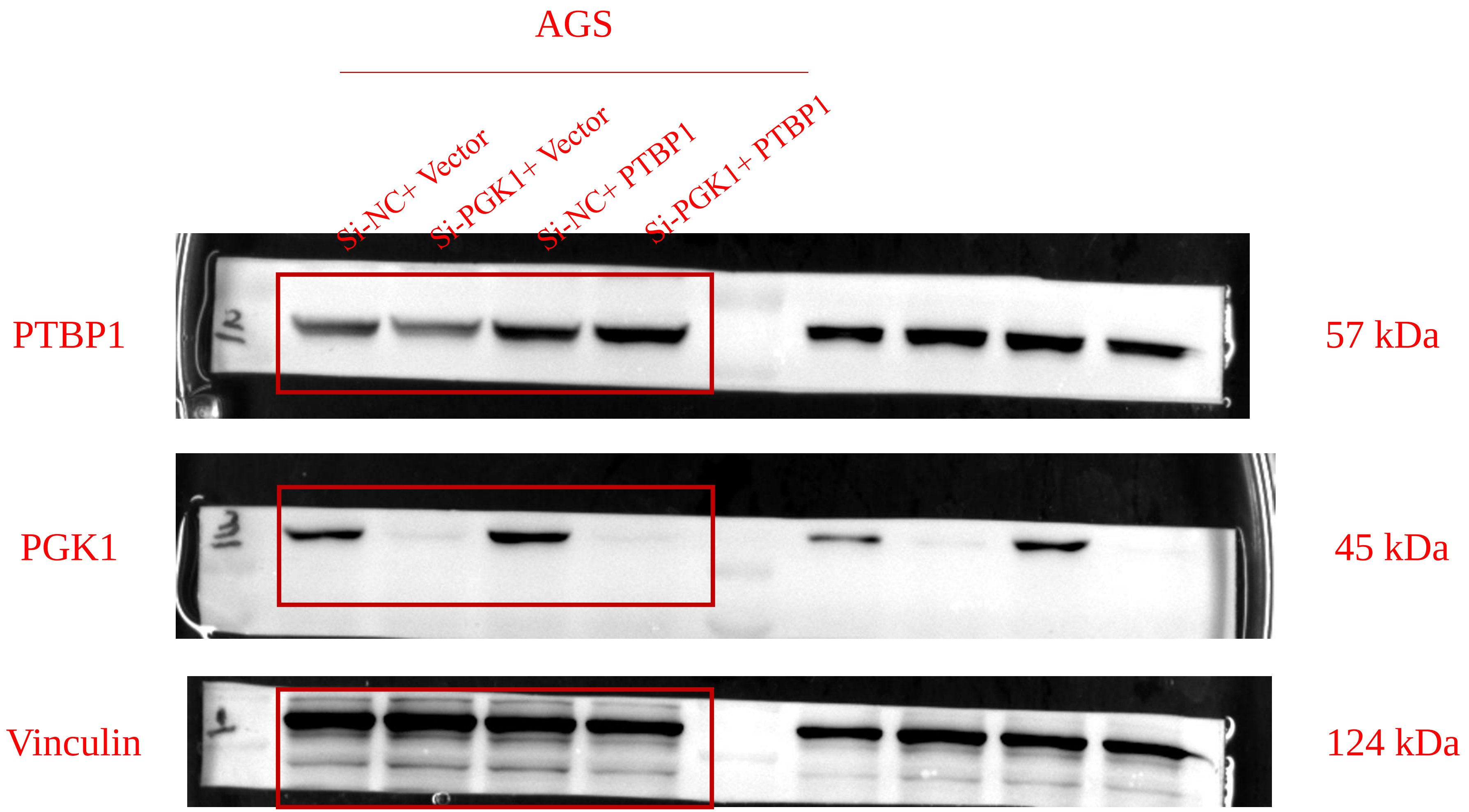

File S1

Uncropped blots for Figure 6A (HGC-27)

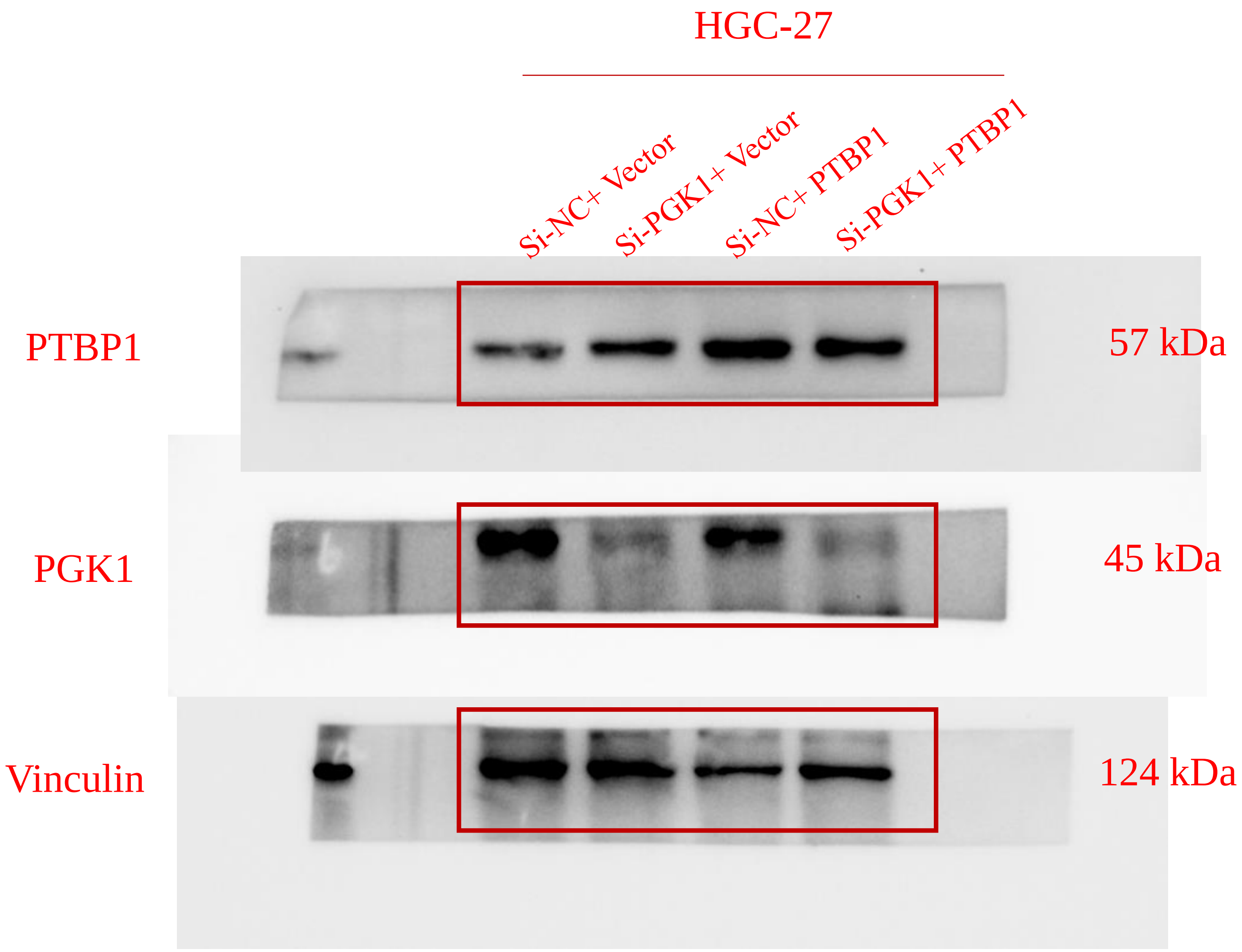

File S1

Uncropped blots for Figure 6C

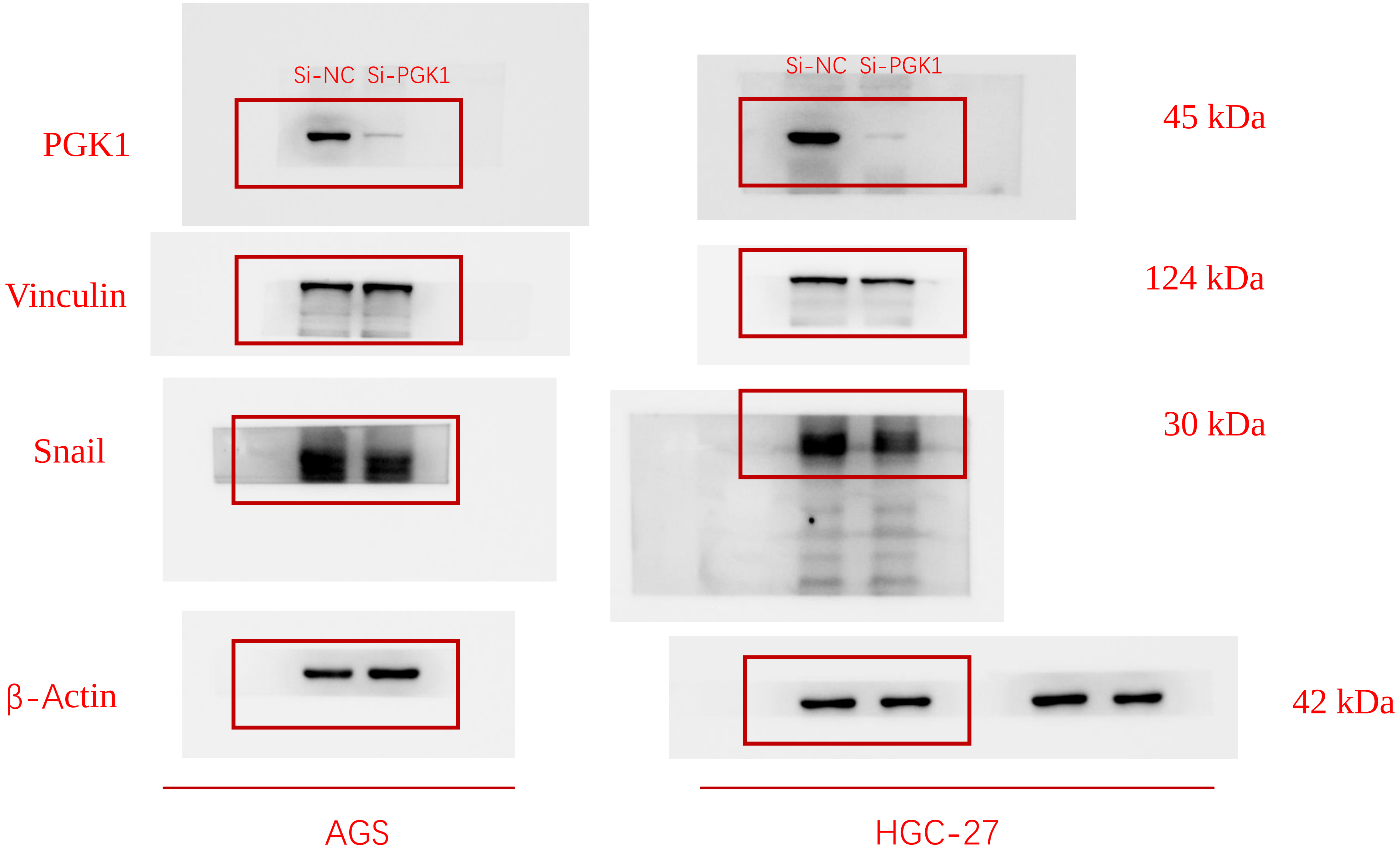

File S1

Uncropped blots for Figure 6D

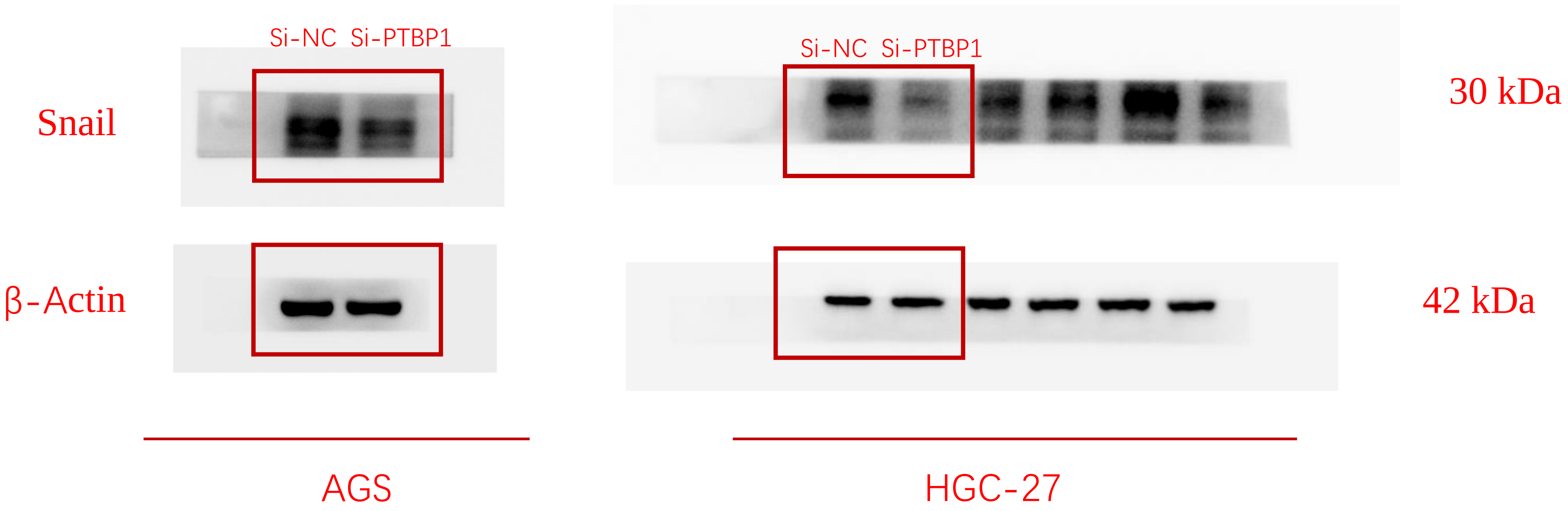

File S1

Uncropped blots for Figure 6E

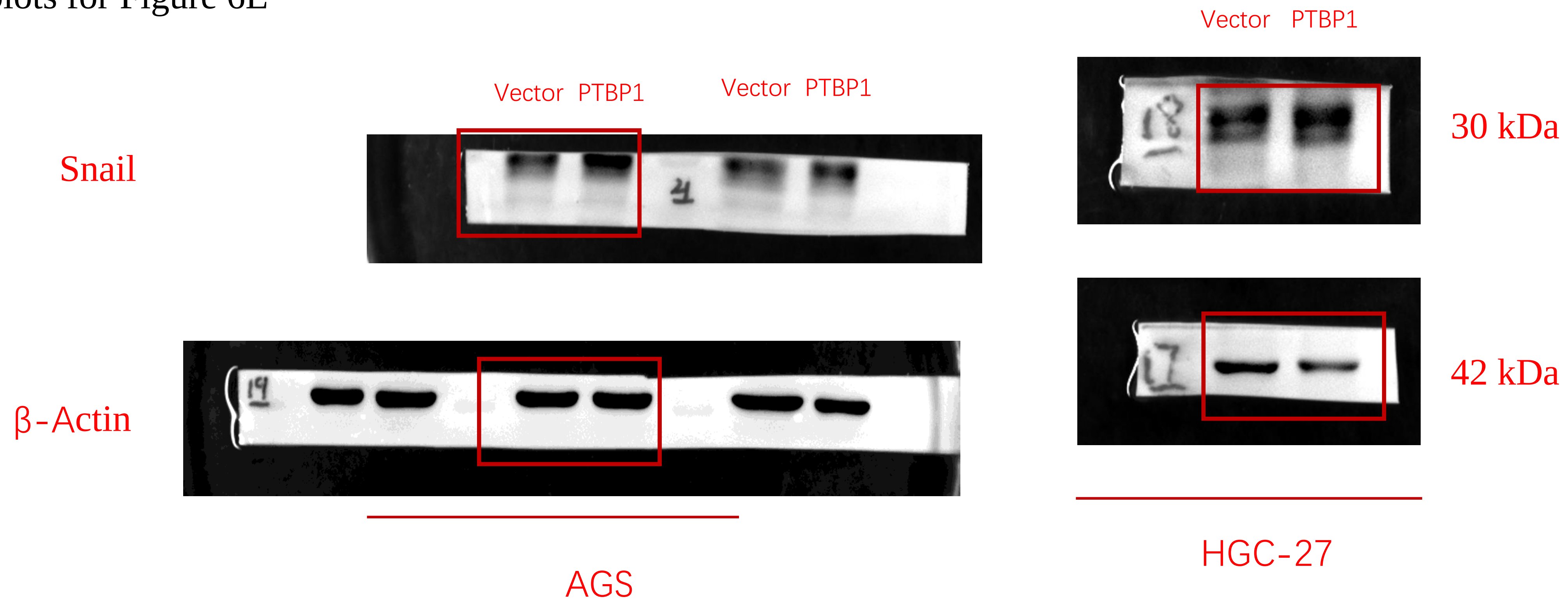

File S1

Uncropped blots for Figure 6F

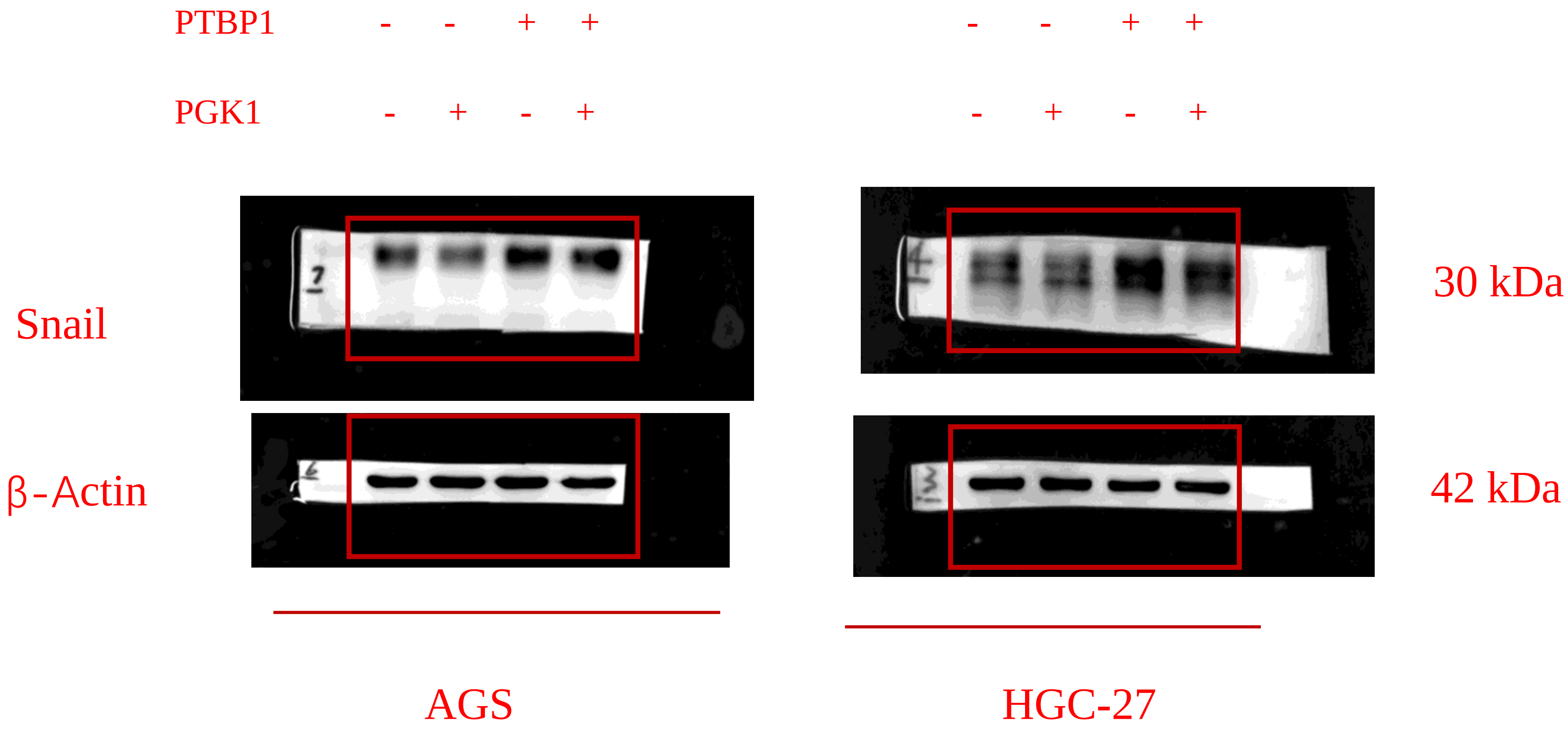

Supplement: Supplementary file 1 [file cells-13-00140-s001.zip › File S1.pdf]
